# Frequently used therapeutic antimicrobials and their resistance patterns on *Staphylococcus aureus* and *Escherichia coli* in mastitis affected lactating cows

**DOI:** 10.1080/23144599.2022.2038494

**Published:** 2022-02-28

**Authors:** Eaftekhar Ahmed Rana, Md Abul Fazal, Mohammad Abdul Alim

**Affiliations:** a Department of Microbiology and Veterinary Public Health, Chattogram Veterinary and Animal Sciences University, Chattogram, Bangladesh; b Department of Pathology and Parasitology, Chattogram Veterinary and Animal Sciences University, Chattogram, Bangladesh

**Keywords:** Antimicrobials, bovine mastitis, MRSA, resistance genes, multidrug resistance

## Abstract

Mastitis is one of the most frequent and costly production diseases of dairy cattle. It is frequently treated with broad-spectrum antimicrobials. The objectives of this work were to investigate the prevalence of *Staphylococcus aureus* and *Escherichia coli*, find out the antimicrobials used in mastitis treatment, and explore the antimicrobial resistance profile including detection of resistance genes. Bacterial species and antimicrobial resistance genes were confirmed by the polymerase-chain reaction. A total of 450 cows were screened, where 23 (5.11%) and 173 (38.44%) were affected with clinical and sub-clinical mastitis, respectively. The prevalence of *S. aureus* was 39.13% (n = 9) and 47.97%(n = 83) while, *E. coli* was 30.43% (n = 7) and 15.60% (n = 27) in clinical and sub-clinical mastitis affected cows, respectively. The highest antimicrobials used for mastitis treatment were ciprofloxacin (83.34%), amoxycillin (80%) and ceftriaxone (76.67%). More than, 70% of *S. aureus* showed resistance against ampicillin, oxacillin, and tetracycline and more than 60% of *E. coli* exhibited resistance against oxacillin and sulfamethoxazole-trimethoprim. Selected antimicrobial resistance genes (*mec*A, *tet*K, *tet*L, *tet*M, *tet*A, *tet*B, *tet*C, *sul*1, *sul*2 and *sul*3) were identified from *S. aureus* and *E. coli*. Surprisingly, 7 (7.61%) *S. aureus* carried the *mec*A gene and were confirmed as methicillin-resistant *S. aureus* (MRSA). The most prevalent resistance genes were *tet*K 18 (19.57%) and *tet*L 13 (14.13%) for *S. aureus*, whereas *sul*1 16 (47.06%), *tet*A 12 (35.29%), *sul*2 11 (32.35%) and *tet*B 7 (20.59%) were the most common resistance genes in *E. coli*. Indiscriminate use of antimicrobials and the presence of multidrug-resistant bacteria suggest a potential threat to public health.

## 1. Introduction

Bovine mastitis remains a highly complex production disease. It poses a great economic challenge for the dairy industries throughout the globe. Diverse groups of pathogens such as bacteria, fungi and mycoplasma have been involved as its causal agents [1]. Around 137 different organisms have been identified as the causal agents of bovine mastitis all over the world [2]. Among them, bacteria are the most common and significant aetiological agent of bovine mastitis occurs in both clinical and sub-clinical forms. However, the most frequent causal agents associated with mastitis are *Staphylococcus aureus* and *Escherichia coli* [3]. Moreover, clinical mastitis is readily detectable based on clinical signs (e.g. pain and/or swelling in the mammary gland) and the presence of abnormal milk (e.g. clots, wateriness). Whereas, in sub-clinical mastitis, the clinical signs and abnormalities in milk are not detectable [4]. The economic losses associated with the clinical and sub-clinical forms of the disease arise from treatment costs, production loss in the form of reduced milk production, genetic potential loss, including culling and death of the affected cows [5].

Mastitis is one of the major reasons for the extensive use of antimicrobials in lactating cows [6–8]. Unfortunately, continuous selective antimicrobial pressure for the treatment and control of bovine mastitis may raise the likelihood of antimicrobial-resistant bacterial strains [7,9]. However, the emergence of these novel antimicrobial-resistant bacteria is one of the principal factors for the failure of recovery from mastitis [10]. So, antimicrobial resistance to mastitic pathogens is a well-documented major challenge for dairy cows’ treatment and management [11]. Furthermore, indiscriminate administration of antimicrobials without susceptibility testing as well as failure to maintain a standard therapeutic dose are considered other significant reasons for treatment failure in mastitis [12]. These misuses of broad-spectrum antimicrobials increase economic losses due to costly treatment processes and also encourage the acquisition of antimicrobial resistance genes from other bacteria in dairy farm environments [7]. Therefore, assessing antimicrobial resistance at the genetic level is of utmost importance to evaluate the determinants of antimicrobial resistance among bacterial pathogens. Moreover, the potential impact of antibiotic residues in milk and transmission of resistant bacteria to humans via the food chain has possible implications for human health.

Exploring the antibiotic resistance pattern of mastitic pathogens is an urgent prerequisite for the implementation of curable therapeutic guidelines and effective control of bovine mastitis. To date, bacteriological and antimicrobial resistance studies of bovine mastitis have not been fully performed in Bangladesh, in particular, the molecular detection of resistance genes is limited. Therefore, considering all the facts, the present study was undertaken to determine the prevalence of *S. aureus* and *E. coli* in clinical and sub-clinical mastitis affected lactating cows and, explore the different classes of antimicrobials administered in the treatment of bovine mastitis. We further aimed to evaluate the current antibiogram trend of *S. aureus* and *E. coli* and also detect the selected antimicrobial resistance genes of these pathogens.

## 2. Materials and methods

### 2.1. Statement of ethics and farm owner consent

The present study was performed according to the ethical guidelines of Chattogram Veterinary and Animal Sciences University (CVASU, Chattogram, Bangladesh). Verbal permission from the dairy farm owners has been taken and minimum discomforts of lactating cows were strictly ensured during screening of the animals and sample collection. All procedures were carried out under the approval of the Ethics Committee of CVASU [Approval no. CVASU/Dir (R&E) EC/2019/41 (2/8)].

### 2.2. Study area and duration

The present cross sectional study was conducted in Shikalbaha and Bandar thana of Chattogram District from November 2018 to June 2020. The area is popularly known as livestock production as well as milk pocket area of Chattogram district. Most of the farmers rear crossbred cows (Holstein-Friesian x Indigenous) are mostly in intensive system for milk production.

### 2.3. Study population and data collection

A total of 30 dairy farms (any farm comprised more than 10 lactating cows) were selected based on previous history of antimicrobials used, mastitis records and presences of clinical mastitis cases in the herd. All the farms were sampled in morning and not more than two farms were sampled in a single day. Pre-tested questionnaires were used for the collection of possible antimicrobials used in clinical and sub-clinical mastitis treatment purposes.

### 2.4. Diagnosis of clinical and sub-clinical mastitis

Clinical mastitis was confirmed based on clinical signs, including hard and inflamed udder, touch to pain, abnormal size and consistency of mammary gland, secretion of abnormal milk (presence of flack, clots, wateriness and pus) and blood-stained milk. At the same time, associated generalized clinical signs such as raises in body temperature, dullness and depression, loss of appetite, and sudden significant reduction of milk production are also considered.

California Mastitis Test was performed for the screening of sub-clinical mastitis in each individual cow. The test was performed according to the protocol described by Rana et al. [13]. Briefly, 2 mL of milk was taken from individual quarters of tested cows in each well of CMT paddles, and equal amounts of CMT reagents were added. The test sample was mixed in a gentle circular motion for 30 seconds. Sub-clinical mastitis was confirmed based on CMT results, the nature of the coagulation and the viscosity of the test mixture. Finally, the test results were interpreted as negative (0 or trace), weakly positive (+); distinctly positive (+ +) and strongly positive (++ +) according to the instructions described by Abebe et al. [14]. A cow was considered positive for sub-clinical mastitis when samples from at least one of the udder quarters tested positive for the California Mastitis Test (CMT) test.

### 2.5. Milk sampling

After the confirmation of clinical and sub-clinical mastitis, the teats of the cow were disinfected with 70% ethyl alcohol. Before sampling, the first squirt of milk was discarded and approximately 5 mL of milk was collected aseptically into a sterile test tube for microbiological analysis. All the samples were finally transferred to the laboratory by maintaining a proper cooling chain using an icebox. All milk samples were kept at room temperature before streaking into the agar plate.

### 2.6. *Isolation, identification and PCR confirmation of* S. aureus *and* E. coli *from mastitic milk*

Bacterial species were identified based on standard bacteriological procedures described by Ali et al. [15] and Gao et al. [16]. In brief, from respective samples, 20 µL of milk was streaked on a 5% bovine blood agar (Oxoid Ltd., Basingstoke, UK) plate and incubated for up to 48 hr at 37°C and the plate was examined every 24 hr interval for optimum growth of desired bacteria. The characteristic appearance of staphylococcus colony is smooth, yellow, pigmented, raised, and with complete or incomplete haemolysis. Suspected colonies were further sub-cultured on Mannitol Salt Agar or MSA (Oxoid Ltd., Basingstoke, UK) and incubated at 37°C for 48 hr. Finally, the Gram’s staining, catalase, and tube coagulase tests were performed on bacterial colonies that fermented MSA. Similarly, suspected *E. coli* colonies (round, thick, moist, smooth, greyish white) were further sub-cultured on MacConkey agar (Oxoid Ltd., Basingstoke, UK) and Eosin methylene blue (EMB) agar and incubated at 37°C for 24 hr. Then, the Gram’s staining, Triple Sugar Iron (TSI) test, Indole, Methyl red, Voges-Proskauer and Citrate utilization (IMViC) tests were performed for biochemical confirmation of *E. coli*.

All primarily isolated *S. aureus* were further confirmed by PCR targeting the 23S rRNA gene described previously by Shome et al. [17] (Table 1) While, *E. coli* species were confirmed by targeting the housekeeping gene (adenylate kinase, *adk*) described by Das et al. [18] (Table 1). All isolates were stored at −80°C using 50% glycerol until further examination [19].

**Table 1.** Primers used for the amplification of *S. aureus* and *E. coli* species and antimicrobial resistance genes

Target genes	Oligonucleotide primer sequences (5̍-3̍)	Reference
*23S* *rRNA*	AGCGAGTCTGAATAGGGCGTTT CCCATCACAGCTCAGCCTTAAC	Shome et al. [17]
*adk*	ATTCTGCTTGGCGCTCCGGG CCGTCAACTTTCGCGTATTT	Das et al. [18]
*mec*A	TCCAGATTACAACTTCACCAGG CCACTTCATATCTTGTAACG	Larsen et al. [22]
*tet*K	GTAGCGACAATAGGTAATAGT GTAGTGACAATAAACCTCCTA	Haubert et al. [23]
*tet*L	TCG TTA GCG TGC TGT CAT TC GTA TCC CAC CAA TGT AGC CG	
*tet*M	GTG GAC AAA GGT ACA ACG AG CGG TAA AGT TCG TCA CAC AC	
*tet*A	GGCGGTCTTCTTCATCATGC CGGCAGGCAGAGCAAGTAGA	Boerlin et al. [24]
*tet*B	CATTAATAGGCGCATCGCTG TGAAGGTCATCGATAGCAGG	
*tet*C	GCTGTAGGCATAGGCTTGGT GCCGGAAGCGAGAAGAATCA	
*sul*1	GTGACGGTGTTCGGCATTCT TCCGAGAAGGTGATTGCGCT	
*sul*2	CGGCATCGTCAACATAACCT TGTGCGGATGAAGTCAGCTC	
*sul*3	GAGCAAGATTTTTGGAATCG CATCTGCAGCTAACCTAGGGCTTTGGA	

### 2.7. Antimicrobial susceptibility testing

All *S. aureus* (92) and *E. coli* (34) bacterial isolates were screened for susceptibility testing using the standard disk diffusion method against 13 different antimicrobial compounds comprising 7 different classes. The antimicrobial panels (Oxoid, Basingstoke, UK) were used, namely: penicillin (10 IU), ampicillin (10 μg), amoxycillin-clavulanic acid (10 μg), cefoxitin (10 μg), ceftriaxone (10 μg), cefaclor (30 μg), ciprofloxacin (10 μg), gentamicin (30 μg), tetracycline (30 μg), erythromycin (15 μg), oxacillin (5 μg), sulfamethoxazole-trimethoprim (1.25 + 23.75 μg), and streptomycin (100 μg). For each isolate, the zone of inhibition around each disk was measured and interpreted as susceptible (S), intermediate (I) and resistant (R) according to the Clinical and Laboratory Standards Institute (CLSI) guidelines [20]. *S. aureus* and *E. coli* isolates that exhibit resistance against ≥3 antimicrobial classes were considered multidrug-resistant (MDR) [21].

### 2.8. Detection of resistance genes

The resistant *S. aureus* isolates (25) to cefoxitin were further screened for the detection of the *mec*A gene by PCR described earlier by Larsen et al. [22] (Table 1) and tetracycline-resistant isolates were confirmed by the presence of the *tet*K, *tet*L and *tet*M genes described by Haubert et al. [23] (Table 1). Also, *E. coli* isolates which showed phenotypic resistance to tetracycline and sulphonamides, were subsequently confirmed by targeting major resistant genes. The *tet*A, *tet*B, *tet*C genes were detected for tetracycline while, *sul*1, *sul*2, *sul*3 genes were detected for sulphonamides by using specific primer sequences previously reported by Boerlin et al. [24] (Table 1). Previously confirmed MRSA strains encoded the *mec*A gene [25], and *E. coli* harboured the tetracycline and sulphonamides resistant genes [18] were used as positive controls, while nuclease-free water was used as negative controls for every PCR reaction.

### 2.9. Statistical analysis

Data obtained from the laboratory and field questionnaires were entered into the Microsoft Excel 2010. The descriptive statistics (percent’s) and the 95% confidence interval of the prevalence values were calculated by the modified Wald method using the Graph Pad Quick Calcs online tool (www.graphpad.com/quickcalcs/). The prevalence of clinical and sub-clinical mastitis was calculated by dividing the number of clinical or sub-clinical mastitis affected cows by the total number of cows tested as the denominator. In addition, the prevalence of *S. aureus* and *E. coli* were enumerated by the number of positive isolates by the total number of mastitis-positive cows sampled as the denominator. Finally, the percentages of resistance genes were determined by the total number of *S. aureus* and *E. coli* positive isolates as the denominator. Furthermore, the heat map and bar diagram were created using Graphpad Prism (version 7).

## 3. Results

### 3.1. Samples

**Figure 1.**
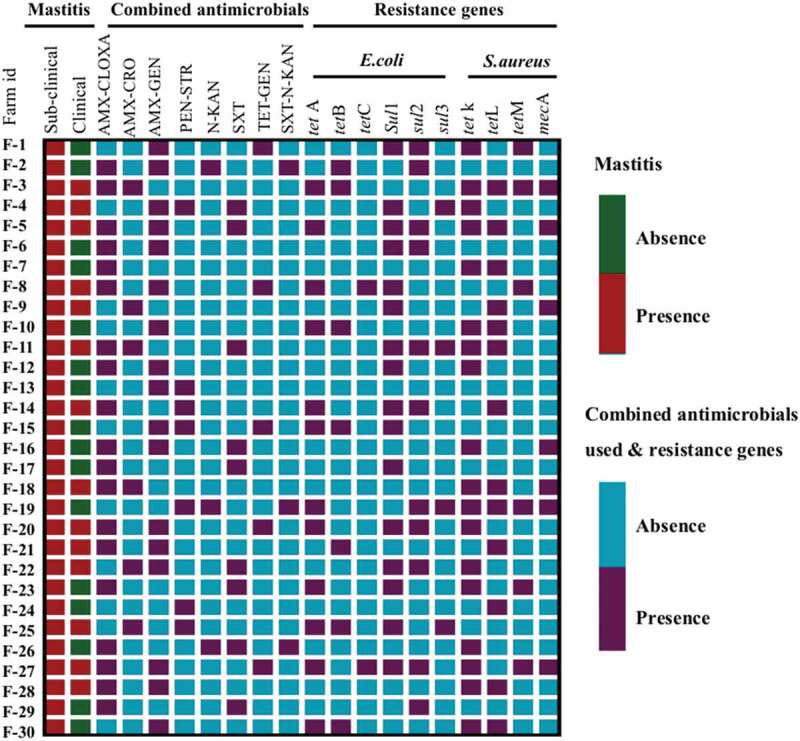
Heat map showing the distribution of types of mastitis circulating in different dairy farms and, various combined antimicrobials used for the treatment of clinical and sub-clinical mastitis, as well as the presence of diverse antimicrobial resistance genes in *S. aureus* and *E. coli* isolates. Each row represents an individual dairy farm. Where, AMX-CLOXA = Amoxycillin- Cloxacillin, AMX-CRO = Amoxycillin- Ceftriaxone, AMX-GEN = Amoxycillin- Gentamicin, PEN-STR = Penicillin- Streptomycin, N-KAN = Neomycin- Kanamycin, SXT = Sulfamethoxazole-Trimethoprim, TET-GEN = Tetracycline- Gentamicin, SXT-N-KAN = Sulfamethoxazole-Trimethoprim- Neomycin- Kanamycin.

In total, 30 dairy farms were screened for clinical and sub-clinical mastitis. Of them, 14 [46.67%, 95% Confidence Interval (CI): 30.23–63.86] farms were positive for clinical mastitis while, all of the farms (100%, 95% CI: 86.53–100) were positive for sub-clinical mastitis (Figure 1). Furthermore, a total of 450 lactating cows (1800 quarters) were randomly screened for identification of sub-clinical mastitis on 30 dairy farms (Table 2). Of them, 173 (38.44%, CI: 34.06 to 43.02) cows were found positive for the CMT test and identified as having sub-clinical mastitis (Table 2). In addition, 23 out of 450 cows (5.11%, 95% CI: 3.27 to 7.57) were found affected with clinical mastitis showing clinical signs and symptoms during the farm visits (Table 2).

**Table 2.** Prevalence of *S. aureus* and *E. coli* in clinical and sub-clinical mastitis affected cows

Total number of dairy farms	Total number of cows screened	Mastitis affected cows (%)	PCR confirmed *S. aureus* (%)	PCR confirmed *E. coli* (%)
30	450 (1800 quarters)	Clinical 23 (5.11%)	9 (39.13)	7 (30.43)
		Sub-clinical 173 (38.44%)	83 (47.97)	27 (15.60)

### 3.2. Grading of bovine clinical and sub-clinical mastitis based of CMT

Among 450 lactating cows, 254 (56.45%, 95% CI: 51.83 to 60.95) animals were found CMT negative, 43 (9.56%, 95% CI: 7.15 to 12.65) were identified as weakly positive (+) and 68 (15.11%, 95% CI: 12.09 to 18.73) were distinctly positive (++) and 62 (13.78%, 95% CI: 10.88 to 17.28) were diagnosed as strongly positive (+++) for sub-clinical mastitis. However, all of the 23 (5.11%, 95% CI: 3.40 to 7.59) clinical mastitis cases were found to be strongly positive (+++) for the CMT.

### 3.3. *Prevalence of* S. aureus *and* E. coli *in clinical and sub-clinical mastitis affected cows*

In total, 196 clinical and sub-clinical mastitic samples were cultured with *S. aureus* being the most prevalent pathogen in both cases, which were 9/23 (39.13%, 95% CI: 22.10 to 59.27) and 83/173 (47.98%, 95% CI: 40.66 to 55.38), respectively (Table 2). Interestingly, all dairy farms were positive for *S. aureus* and associated with bovine mastitis. Among the 92*S. aureus* isolates, 34 (36.96%, 95% CI: 27.79 to 47.17) were found to be positive for coagulase test and the remaining 58 (63.04%, 95% CI: 52.83 to 72.21) were identified as coagulase test negative. The prevalence of *E. coli* was found to be 7/23 (30.43%, 95% CI: 15.41 to 51.06) and 27/173 (15.61%, 95% CI: 10.90 to 21.81) in clinical and sub-clinical mastitis affected cows, respectively (Table 2).

### 3.4. Frequency of antimicrobial used in dairy farm

**Figure 2.**
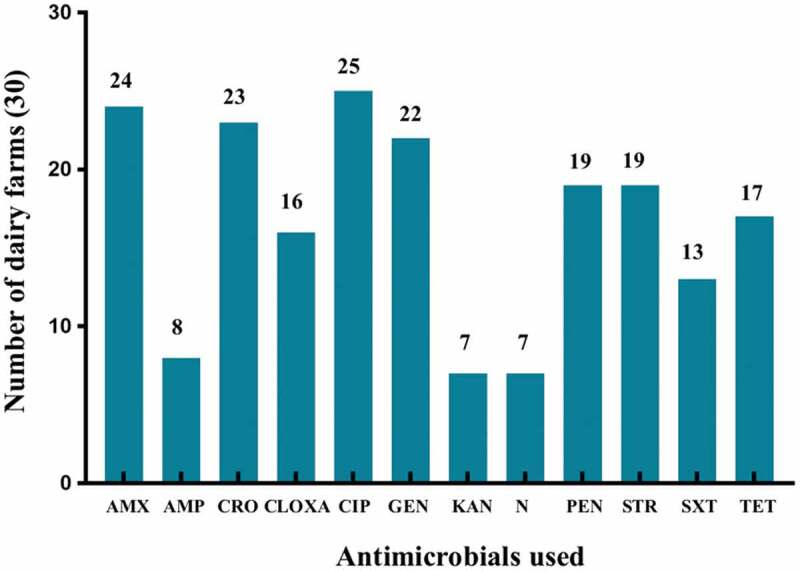
Diverse group of antimicrobials used in dairy farms for the treatment of clinical and sub-clinical mastitis. Where, AMX = Amoxycillin, AMP = Ampicillin, CRO = Ceftriaxone, CLOXA = Cloxacillin. CIP = Ciprofloxacin, GEN = Gentamicin, KAN = Kanamycin, N = Neomycin, PEN = Penicillin, STR = Streptomycin, SXT = Sulfamethoxazole-trimethoprim, TET = Tetracycline.

We found that 12 different kinds of antimicrobials were used in 30 different dairy farms. Among them, the highest antimicrobials used for mastitis treatment were ciprofloxacin in 25 (83.33%) farms; amoxycillin in 24 (80%) farms; ceftriaxone in 23 (76.67%) farms, and gentamicin in 22 (73.33%) farms (Figure 2). Besides, penicillin and streptomycin were used in 19 (63.33%) farms, tetracycline in 17 (56.67%) farms, cloxacillin in 16 (53.33%) and sulfamethoxazole-trimethoprim in 13 (43.33%) farms at the second highest level. The frequency of neomycin 7 (23.33%), and kanamycin 7 (23.33%) usage in the dairy farms were minimal (Figure 2).

### 3.5. Combination of antimicrobial used for mastitis treatment

Interestingly, all of the dairy farms (n = 30) used more than 3 antimicrobial classes for mastitis treatment, while 23 (76.67%) farms administered different combined antimicrobials in different combinations (Figure 1). Among them, a maximum of 19 (63.33%) farms used amoxycillin-cloxacillin and 17 (56.67%) farms used amoxycillin-gentamicin combined antimicrobials for mastitis treatment. There were 7 (23.33%) farms used single antimicrobials for therapeutic purposes (Figure 1).

### 3.6. Antimicrobial susceptibility profiles

All the *S. aureus* and *E. coli* isolates were found to be multi-drug resistant (MDR) (i.e. resistant to ≥ 3 antimicrobial classes). Among the *S. aureus* isolates, the highest resistance was observed for tetracycline (76.09%), oxacillin, and ampicillin (70.65%) (Table 3). More than 58% of the isolates exhibited resistance against erythromycin, amoxycillin-clavulanic acid and streptomycin. In the case of *E. coli*, this exhibits the highest resistance against oxacillin (64.71%) and ampicillin (58.82%) antibiotics. More than 70% of *E. coli* isolates were sensitive to ceftriaxone, ciprofloxacin and cefaclor (Table 3).

**Table 3.** Antimicrobial sensitivity profile of *S. aureus* and *E. coli* in clinical and sub-clinical mastitis affected cows

Organism	Antimicrobial susceptibility	AUG	AMP	CRO	CEC	FOX	CIP	ERY	GEN	OXA	PEN	STR	SXT	TET
*S. aureus* (92)	Sensitive (%)	38 (41.30)	27 (29.35)	65 (70.65)	48 (52.17)	67 (72.83)	60 (65.22)	38 (41.30)	33 (35.87)	27 (29.35)	44 (47.83)	38 (41.30)	48 (52.17)	22 (23.91)
	*Resistant (%)	54 (58.70)	65 (70.65)	27 (29.35)	44 (47.83)	25 (27.17)	32 (34.78)	54 (58.70)	59 (64.13)	65 (70.65)	48 (52.17)	54 (58.70)	44 (47.83)	70 (76.09)
*E. coli* (34)	Sensitive (%)	18 (52.94)	14 (41.18)	25 (73.53)	24 (70.59)	23 (67.65)	25 (73.53)	26 (76.47)	23 (67.65)	12 (35.29)	19 (55.88)	23 (67.65)	13 (38.24)	19 (55.88)
	*Resistant (%)	16 (47.06)	20 (58.82)	9 (26.47)	10 (29.41)	11 (32.35)	9 (26.47)	8 (23.53)	11 (32.35)	22 (64.71)	15 (44.12)	11 (32.35)	21 (61.76)	15 (44.12)

*All intermediately resistant isolates are considered as susceptible. Where, AUG = Amoxycillin-Clavulanic acid, AMP = Ampicillin, CRO = Ceftriaxone, CEC = Cefaclor, FOX = Cefoxitin, CIP = Ciprofloxacin, ERY = Erythromycin, GEN = Gentamicin, OXA = Oxacillin, PEN = Penicillin, STR = Streptomycin, SXT = Sulfamethoxazole-Trimethoprim, TET = Tetracycline.

### 3.7. *Antimicrobial resistance genes profile of* S. aureus *and* E. coli

A total of 10 resistance genes including *mec*A, *tet*K, *tet*L, and *tet*M, were evaluated for *S. aureus*. The *tet*A, *tet*B, *tet*C, *sul*1, *sul*2, and *sul*3, were screened for *E. coli* isolates. Among the *S. aureus* isolates, 7/92 (7.61%) encoded the *mec*A gene, which is classified as methicillin-resistant *S. aureus* (MRSA) and 19.57% (18/92) tested isolates carried *tet*K gene (Figure 1) (Table 4). In the case of *E. coli* isolates, the most prevalent antimicrobial resistance genes were *tet*A 12/34 (35.29%) and *tet*B 7/34 (20.59%) encoding resistant to tetracycline followed by *sul*1 16/34 (47.06%) and *sul*2 11/34 (32.35%), encoding resistant to sulphonamides (Figure 1) (Table 4).

**Table 4.** Prevalence of different antimicrobial resistance genes encoded by *S. aureus* and *E. coli.*

Organism	Resistance genes	Prevalence
*S. aureus* (92)	*mec*A	7 (7.61%)
	*tet*K	18 (19.57%)
	*tet*L	13 (14.13%)
	*tet*M	6 (6.52%)
*E. coli* (34)	*tet*A	12 (35.29%)
	*tet*B	7 (20.59%)
	*tet*C	2(5.88%)
	*sul*1	16 (47.06%)
	*sul*2	11(32.35%)
	*sul*3	4(11.76%)

## 4. Discussion

In the present study, the overall prevalence of sub-clinical mastitis at cow level was 38.44% and the smallest proportion (5.11%) was suffered from clinical mastitis. This finding was consistent with the previous reports of Ramírez et al. [11,26]. The prevalence of clinical and sub-clinical mastitis was slightly higher in Eastern Ethiopia, which was 12.5% and 51.8%, respectively [27]. The occurrence of mastitis in lactating cows varies due to the geographical location, breed, age, stages of lactation, status of the udder, number of parity, immunity, management, hygiene, and milking practices in dairy farms [26,28]. However, the predominant sub-clinical mastitis in the current study area is an alarming problem that not only causes economic losses through reducing milk production but also adversely affects the human food chain.

Both *S. aureus* and *E. coli* are the predominant causal agents of bovine clinical and sub-clinical mastitis all over the world [23] including Bangladesh. However, in both clinical and sub-clinical cases, the most frequently isolated organism was *S. aureus*, which represented 39.13% and 47.98%, respectively. The isolation rate was closely similar to the studies conducted in Italy (41%) [29] and slightly higher in Brazil (56%) [30]. Among *S. aureus*, 36.96% was found to be coagulase positive, which was closely similar to the previous findings [31]. These coagulase positive *S. aureus* strains are most frequently associated with contagious mastitis of bovine, caprine and ovine species due to the harbouring of multiple virulence factors such as Staphylococcal Protein-A, the coagulase enzyme, clumping factors, haemolysins, proteases and gelatinases [13,23,32].

Moreover, *E. coli* also causes environmental fatal mastitis and their frequency varies according to farm management, hygienic practices, and the presence of virulence factors. Surprisingly, any type of mastitis in the mammary gland always first attempts to be treated with antimicrobials to reduce the fatal consequence, especially to control the contagious infections [6]. In the present study, we found that all of the dairy farms administered different categories of antimicrobials (penicillin, β-lactams, fluoroquinolones, tetracyclines, aminoglycoside and sulphonamide) ranging from narrow to broad-spectrum to treat the clinical and sub-clinical mastitis. Another significant concern was the high number of dairy owners who used combined antimicrobials in a combination of different classes for the treatment of clinical and sub-clinical mastitis. Unfortunately, the widespread and indiscriminate administration of antimicrobials induces the development of resistance among mastitis bacteria [33]. The repetitive exposure of a similar or wide range of antimicrobials for the treatment of chronic/sub-clinical mastitis play a key trigger for antimicrobial resistance and contribute to acquire resistant genes. Our results demonstrated that all isolates were multidrug-resistant and showed resistance to three or more classes of antimicrobials [21]. This finding indicates the emergence of antimicrobial resistance in *S. aureus* and *E. coli* from masitic milk samples in Bangladesh. Moreover, the number of resistant bacteria evolved in increasing trend from bovine mastitis cases that have potential chance to spread in the human food chain and poses a zoonotic burden in environment.

Since the screening of antimicrobial resistance genes, both *S. aureus* and *E. coli* isolates harboured multiple types of resistance genes. According to different mastitis research, it is focused that β-lactams antimicrobials are most frequently used in both parental and intramammary routes in lactating cows for treatment of mastitis infection [34,35]. In the present study, high antimicrobial resistance frequency to the β-lactams penicillin (52.17%), amoxycillin-clavulanic acid (58.70%), ampicillin (70.65%), oxacillin (70.65%) was observed, which is closely supported by previous findings of Priyantha et al. [34]; Haubert et al. [23]. According to previous findings, *S. aureus* and *E. coli* resistance to penicillin, ampicillin, amoxycillin, and oxacillin is the most common type of antimicrobial resistance among mastitis-causing bacteria [9,11,13,34]. The high resistance frequency of *E. coli* against sulfamethoxazole-trimethoprim (61.76%) was reported in several previous studies [9,34]. We noted that *S. aureus* and *E. coli* displayed a high proportion of susceptibility to ceftriaxone (70.65% and 73.53%) and ciprofloxacin (65.22% and 73.53%) which was consistent with the findings of previously published reports [9,13,15]. The high rate of susceptibility to ceftriaxone and ciprofloxacin might be due to less exposure in dairy farm environments as well as the broad-spectrum nature of these antimicrobials.

Interestingly, in the present study 7 (7.61%) *S. aureus* isolates carried the *mec*A gene and all MRSA isolates exhibit resistance to cefoxitin, oxacillin and penicillin, and these findings were almost similar to the previous studies by Rana et al. [13]; Chajecka-Wierzchowska et al. [36]. The *mec*A gene is extensively found in staphylococci isolates which confer the resistance to β-lactams antimicrobials and this resistant gene arises from a chromosomally integrated mobile genetic element namely staphylococcal cassette chromosome *mec* (SCC*mec*) [37]; which encodes modified penicillin-binding protein (PBP). Although few studies have reported the presence of the *mec*A gene in *S. aureus* at low levels in bovine and caprine mastitis [13,38,39], but the widespread presence of it creates a potential challenge for clinical management of mastitis. Moreover, these resistant isolates are readily transferable from livestock to humans that make a potential zoonotic burden. The MSRA isolates constantly display resistance against a wide range of broad spectrum antimicrobial agents, including all β-lactams antimicrobials (penicillin, methicillin, oxacillin, cefoxitin, amoxycillin-clavulanic acid, amoxycillin-sulbactam), quinolones, tetracycline, macrolides and chloramphenicol [22,32,40].

Fluoroquinolones, tetracyclines and sulphonamides are broad-spectrum antimicrobial agents administered in mastitis cows for clinical recovery of infection [36]. We noted that both *S. aureus* and *E. coli* showed 76.09% and 44.12% resistance against tetracycline, respectively. Moreover, the presence of *tet*A, *tet*B, *tet*C, *tet*K, *tet*L and *tet*M genes conferred the efflux proteins or ribosomal protection proteins that trigger the high resistance against tetracycline antibiotics. The presence of a tetracycline resistance gene among *S. aureus* and *E.coli* from clinical and sub-clinical mastitis affected cows has been reported in several previous studies [23,28,34]. In the present study, *S. aureus* and *E. coli* harboured *tet*K and *tet*A genes at the highest frequencies, which were 19.57% and 35.29%, respectively. However, the presence of other tetracycline resistance genes is found at a lower rate in bovine mastitis cases and these findings was in agreement with previous studies of Ashraf et al. [28]; Haubert et al. [23]. Furthermore, several studies discloses that the widespread distribution of tetracycline resistance determinants among different organisms is largely due to the acquisition or encoding of mobile genetic elements like readily transferable conjugative plasmids or transposons [41].

In the current study, the sulphonamide resistance genes that inhibit the folate pathways like *sul*1 (47.06%), *sul*2 (32.35%), and *sul*3 (11.76%) were detected in *E. coli* isolates. The frequencies of résistance genes are significantly higher than previous studies by Frey et al. [42] who described only 5% isolates carrying sulphonamide resistance genes in bovine mastitis cases. However, the presence of sulphonamide resistance genes in *E. coli* at higher rates in raw milk and milk products, which may originate from clinical or sub-clinical mastitis, affected lactating cows in the dairy farm [30].

Due to resource limitations, sulphonamide resistance genes for *S. aureus*, was not detected in the present study. The emergence of multidrug-resistant (MDR) organisms in dairy herds is a serious threat that is resulting in significant increases in veterinary as well as medication costs for treatment and management of bovine mastitis. Generally, the MDR phenomenon is significantly triggered by the spontaneous and irrational use of widespread antimicrobials and acquiring of multiple antimicrobial resistance genes through cross-transmission, mutation or from environments [21,32,41]. Therefore, it is an urgent need to monitor and control the irrational use of antimicrobial agents and the exemplary mastitis treatment guidelines should be based on the laboratory reports of the antimicrobials sensitivity test.

## 5. Conclusion

This study demonstrated that sub-clinical type mastitis is highly prevalent in dairy farms and *S. aureus* is confirmed as the most prevalent pathogen associated with both clinical and sub-clinical bovine mastitis. Alarmingly, all dairy farms used broad-spectrum antimicrobials, especially ciprofloxacin, amoxycillin, and ceftriaxone for the treatment of bovine mastitis. Moreover, the presence of MRSA strains and the encoding of different resistance genes on *S. aureus* and *E.coli* isolates in mastitic cows is an alarming report and might pose a serious challenge to the clinical management of bovine mastitis. Therefore, it is of utmost importance to conduct routine surveillance programs to regulate the use of antimicrobial agents and detect the potential transfer of antimicrobial resistance genes on dairy farms.

## Data Availability

The authors confirm that the data supporting the findings of this study are available within the article.
